# ASC, IL-18 and Galectin-3 as Biomarkers of Non-Alcoholic Steatohepatitis: A Proof of Concept Study

**DOI:** 10.3390/ijms21228580

**Published:** 2020-11-13

**Authors:** Brianna Cyr, Robert W. Keane, Juan Pablo de Rivero Vaccari

**Affiliations:** 1 Department of Neurological Surgery and The Miami Project to Cure Paralysis, Miller School of Medicine, University of Miami, Miami, FL 33136, USA; bxc205@miami.edu (B.C.); rkeane@miami.edu (R.W.K.); 2 InflamaCORE, LLC, Miami, FL 33156, USA; 3 Department of Physiology and Biophysics, University of Miami Miller School of Medicine, Miami, FL 33136, USA; 4 Center for Cognitive Neuroscience and Aging, University of Miami Miller School of Medicine, Miami, FL 33136, USA

**Keywords:** NASH, inflammasome, biomarkers, inflammation, ASC

## Abstract

Non-alcoholic steatohepatitis (NASH) is a severe form of non-alcoholic fatty liver disease that is growing in prevalence. Symptoms of NASH become apparent when the disease has progressed significantly. Thus, there is a need to identify biomarkers of NASH in order to detect the disease earlier and to monitor disease severity. The inflammasome has been shown to play a role in liver diseases. Here, we performed a proof of concept study of biomarker analyses (cut-off points, positive and negative predictive values, receiver operating characteristic (ROC) curves, and likelihood ratios) on the serum of patients with NASH and healthy controls on apoptosis-associated speck-like protein containing a caspase recruitment domain (ASC), interleukin (IL)-18, Galectin-3 (Gal-3), and C-reactive protein (CRP). ASC, IL-18, and Gal-3 were elevated in the serum of NASH patients when compared to controls. The area under the curve (AUC) for ASC was the highest (0.7317) with an accuracy of 68%, followed by IL-18 (0.7036) with an accuracy of 66% and Gal-3 (0.6891) with an accuracy of 61%. Moreover, we then fit a stepwise multivariate logistic regression model using ASC, IL-18, and Gal-3 to determine the probability of patients having a NASH diagnosis, which resulted in an AUC of 0.71 and an accuracy of 79%, indicating that combining these biomarkers increases their diagnostic potential for NASH. These results indicate that ASC, IL-18, and Gal-3 are reliable biomarkers of NASH and that combining these analytes increases the biomarker potential of these proteins.

## 1. Introduction

Non-alcoholic fatty liver disease (NAFLD) is a common liver disease, which ranges from steatosis to non-alcoholic steatohepatitis (NASH), the most severe type [1]. A patient with steatosis may eventually progress to NASH if no treatment is initiated. NASH is characterized by steatosis, inflammation, and a characteristic pattern of hepatocellular injury [1]. NASH may progress to cirrhosis, cancer, and eventually the patient may require a liver transplant [1]. The prevalence of NASH has grown over the last few decades, and it is predicted to increase in frequency. For instance, cases in 2016 were estimated at 17.32 million cases in the United States, and it is estimated that the number of cases by the year 2030 will rise by 56% [2]. Currently, the diagnosis of NASH is made by a liver biopsy. Due to the invasive nature of this diagnostic method and the severity of the disease, it is imperative to develop a less invasive test using blood biomarkers in order to diagnose and monitor the disease with more ease and accuracy.

Galectin-3 (Gal-3) is a lectin protein that binds galactose-containing glycoproteins, and it is involved in the pathology of fibrosis in a number of organs, including the liver [3]. Furthermore, Gal-3 expression is associated with increased severity of steatosis and NASH. Gal-3 ablation protects mice from high fat diet-induced NASH when compared to wildtype mice [4]. Moreover, Gal-3 deficient mice present with milder liver disease when compared to wildtype controls. However, there have also been studies that show that when compared to wildtype mice, Gal-3 knockout mice promote hepatic injury when fed a choline deficient amino acid-defined (CDAA) diet [5] and steatosis when fed a high fat diet [6]. Each of these diets are used to model NAFLD in rodents. Despite the contrasting evidence on the role of Gal-3 in NASH, Gal-3 inhibitors are currently being developed as potential treatments for NASH [7].

A primary characteristic of NASH that distinguishes it from other NAFLDs is inflammation [2]. Following liver injury, an inflammatory response is initiated that is mediated in part by the pro-inflammatory cytokine interleukin (IL)-1β that is released from cells in the liver [8]. Active IL-1β is released via the inflammasome, a multiprotein complex that typically contains a NOD-like receptor (NLR), an adaptor protein known as apoptosis-associated speck-like protein containing a caspase recruitment domain (ASC), and inflammatory caspase-1 [9]. When the inflammasome is activated, it processes IL-1β and IL-18 into their respective active forms to then spread inflammation to other tissues and cells.

Inflammasomes are present in hepatocytes [10], liver stellate cells [11], and sinusoidal endothelial cells [12]. The NLRP3 inflammasome, in particular, plays a role in the progression of NASH [13]. In wildtype mice, the CDAA diet induces hepatic steatosis. However, when the same diet is given to NLRP3 knockout mice, they are protected from liver injury and fibrosis while NLRP3 knock-in mice showed severe liver inflammation and early signs of fibrosis. In addition, mRNA expression levels of NLRP3, ASC, caspase-1, pro-IL-1β, and pro-IL-18 are upregulated in the liver of NASH patients compared to healthy controls [10,13].

Inflammasome may lead to pyroptosis, which causes cells to release their contents, including inflammasome proteins, following cell death [9], and thus, it contributes to the spread of the inflammatory response. This release of inflammasome proteins makes inflammasome proteins promising biomarkers of inflammation. Our studies have shown inflammasome signaling proteins as inflammatory biomarkers of brain injury [14], stroke [15], Alzheimer’s disease [16], mild cognitive impairment [16], depression [17], multiple sclerosis [18], and psoriasis [19]. In this paper, we extend our previous biomarker studies by examining the protein levels of ASC, C-reactive protein (CRP), IL-18, and Gal-3 as potential biomarkers of NASH. We conducted receiver operating characteristic (ROC) analysis with associated sensitivity and specificity as well as calculated cut-off points for the diagnosis of NASH.

## 2. Results

### 2.1. ASC, IL-18, and Gal-3 Are Elevated in Serum of NASH Patients

**Figure 1:**
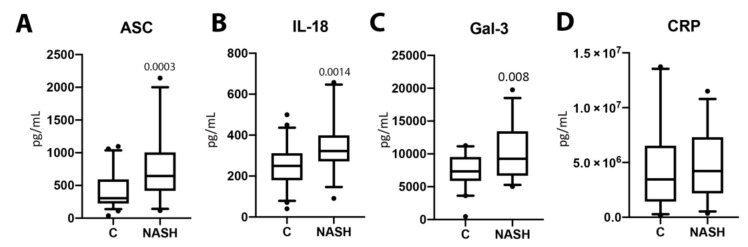
The concentration of inflammasome proteins is increased in NASH patient serum. The protein concentration levels in pg/mL of ASC (**A**), IL-18 (**B**), Gal-3 (**C**), and CRP (**D**) in the serum of aged-matched controls and NASH patients. *N* = ASC: 53 controls, 32 NASH; IL-18: 58 controls, 31 NASH; Gal-3: 39 controls, 32 NASH; and CRP: 33 controls, 31 NASH. Box and whiskers are shown for the 5th and 95th percentile. C: Control; NASH: Non-alcoholic Steatohepatitis; ASC: Apoptosis-Associated Speck-like Protein Containing a Caspase Recruitment Domain; IL-18: Interleukin-18; Gal-3: Galectin-3; CRP: C-Reactive Protein.

Serum samples from NASH patients and normal age-matched controls were analyzed for the protein levels of ASC (*p* = 0.0003, Figure 1A), IL-18 (*p* = 0.0014, Figure 1B), Gal-3 (*p* = 0.008, Figure 1C), and CRP (*p* = 0.4385, (Figure 1D). The protein expression levels for ASC, IL-18, and Gal-3 were significantly higher in the serum of NASH patients when compared to healthy controls. However, CRP protein expression levels were not significantly different between groups. These data suggest an involvement of ASC, IL-18, and Gal-3 in the pathology of NASH.

### 2.2. ASC Is a Potential Serum Biomarker of NASH

**Figure 2:**
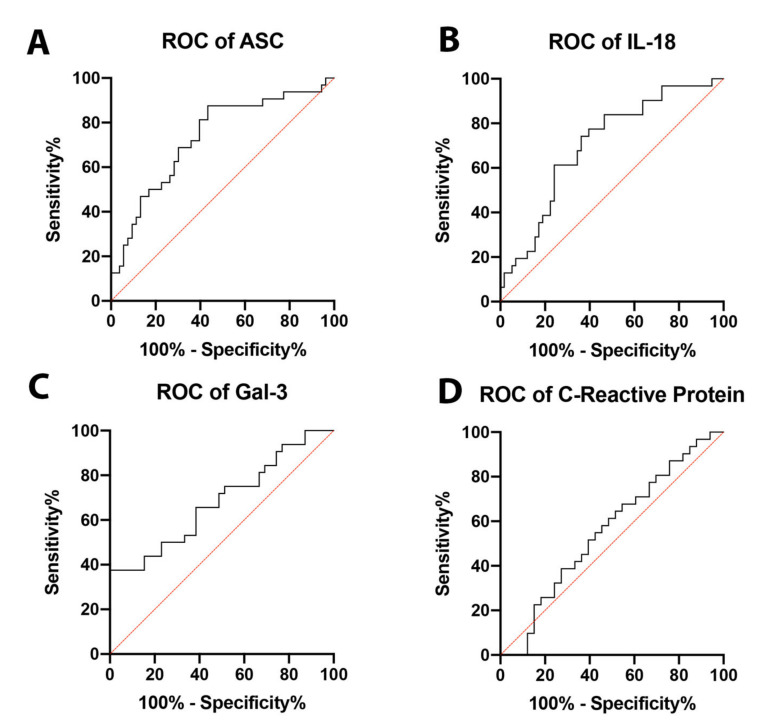
ROC curves for analyzed proteins. ROC curves for ASC (**A**), IL-18 (**B**), Gal-3 (**C**), and CRP (**D**) from the serum of aged-matched controls and NASH patients. *N* = ASC: 53 controls, 32 NASH; IL-18: 58 controls, 31 NASH; Gal-3: 39 controls, 32 NASH; and CRP: 33 controls, 31 NASH. C: Control; NASH: Non-alcoholic Steatohepatitis; ASC: Apoptosis-Associated Speck-like Protein Containing a Caspase Recruitment Domain; IL-18: Interleukin-18; Gal-3: Galectin-3. ROC: Receiver Operating Characteristics.

**Figure 3:**
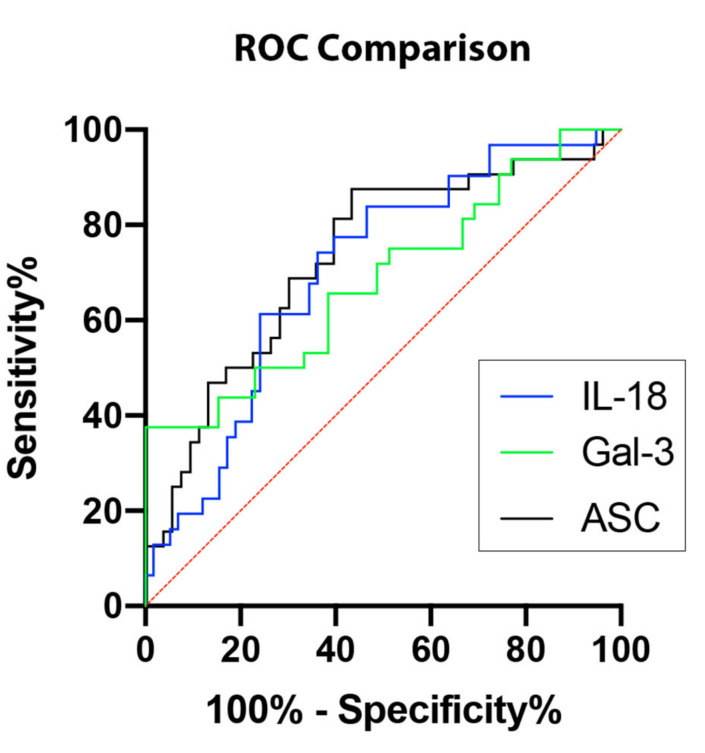
Inflammasome proteins as potential biomarkers for NASH. Comparison of ROC curves for ASC (black), IL-18 (blue), and Gal-3 (green). *N* = ASC: 53 controls, 32 NASH; IL-18: 58 controls, 31 NASH; Gal-3: 39 controls, 32 NASH. ROC: Receiver Operating Characteristics.

In order to establish whether ASC, IL-18, and Gal-3 proteins could be considered as reliable biomarkers of NASH, the area under the curve (AUC) was determined for ASC (Figure 2A), IL-18 (Figure 2B), Gal-3 (Figure 2C), and CRP (Figure 2D). The AUC of each protein analyzed was compared to each other (Figure 3). ASC had the highest AUC of 0.7317 (*p* = 0.0004) (Table 1), IL-18 had an AUC of 0.7036 (*p* = 0.0016) (Table 1), and Gal-3 had an AUC of 0.6891 (*p* = 0.0064) (Table 1). The cut-off point for ASC was 394.9 pg/mL with 81% sensitivity and 60% specificity, the IL-18 cut-off point was 269.2 pg/mL with 77% sensitivity and 60% specificity, and for Gal-3, the cut-off point was 7120 pg/mL with 75% sensitivity and 49% specificity (Table 2); thus, indicating that ASC, IL-18, and Gal-3 are reliable biomarkers of NASH.

### 2.3. Logistic Regression between ASC, IL-18, and Gal-3

To predict the probability that either ASC, IL-18, and Gal-3 contribute to the pathology of NASH, we ran binomial logistic regression models for the probability of explaining the diagnosis of NASH. Accordingly, three univariate models were tested using each analyte that showed statistically significant difference between controls and NASH patients. All three analytes presented AUC values greater than 0.65 (ASC (0.82), IL-18 (0.74), and Gal-4 (0.69)) and a Hosmer–Lemeshow p-value greater than 0.05 (ASC (0.29), IL-18 (0.34), and Gal-4 (0.12)) (Table 3)

Then, the analytes were used to fit a multivariate binominal logistic regression model (Table 4). Accordingly, the multivariate logistic regression model presented the following metrics: AUC 0.71, McFadden Pseudo-R^2^ 0.35, Hosmer–Lemeshow *p*-value: 0.49, AIC: 70.37, and BIC: 79.36. Overall, this model was 79% accurate with an 81% sensitivity, a 60% specificity, 68% PPV, and 87% NPV.

## 3. Discussion

Here, we provide evidence that ASC, IL-18, and Gal-3 potentially play a role in the pathology of NASH and may serve as reliable biomarkers of NASH. All three proteins were elevated in serum of NASH patients compared to age-matched controls. In this study, we calculated AUC values for ASC, IL-18, and Gal-3. We show that ASC is the most reliable biomarker with the highest AUC (0.7317), followed by IL-18 (0.7036) and Gal-3 (0.6891). To further predict whether ASC, IL-18, and Gal-3 may serve as biomarkers of NASH pathology, we ran a binomial logistical regression. Our analyses indicate that the odds of a NASH diagnosis increased with increased protein levels of ASC (exponentiated coefficient: 1.0052), IL-18 (exponentiated coefficient: 1.0083), and Gal-3 (exponentiated coefficient: 1.0002) in serum. The accuracy of each individual analyte for the diagnosis of NASH corresponded to 68, 66, and 61%, respectively. However, when all three proteins were combined in a multivariate logistic regression model, the accuracy increased to 79%.

NAFLD is an umbrella term for a group of fatty liver diseases not induced by alcohol consumption. It encompasses steatosis as well as NASH. Steatosis (also known as non-alcoholic fatty liver) is characterized by fat accumulation in more than 5% of hepatocytes, and it is the least severe form of NAFLD, whereas NASH is characterized by inflammation and hepatocellular injury in addition to steatosis and is the most severe form of NAFLD [1]. NASH is likely to progress to fibrosis, cirrhosis, and even hepatocellular carcinoma [20].

Gal-3 is implicated in the progression of fibrosis of many different organs, including the liver [3], and has been implicated in the regulation of metabolic disorders including obesity and diabetes [21,22]. Gal-3 has been found to mediate inflammation and injury in acetaminophen-induced hepatotoxicity [23]. There is contradicting reports as to how Gal-3 affects NASH pathology. For instance, ablation of Gal-3 seems to protect mice from NASH development and severity in one study [4]. Alternatively, other studies have shown that Gal-3 ablation increases hepatocellular injury [5] and steatosis [6]. A fourth study suggests that Gal-3 has multiple roles in NASH, with CD68/Gal-3^+^ cells decreasing with severity of steatosis and NASH and α-smooth muscle actin/Gal-3^+^ cells increasing with severity of fibrosis [24]. Additionally, a Gal-3 inhibitor recently underwent a phase 2b clinical trial, but there were no significant effects on fibrosis or portal hypertension [7]. Consistent with a potential role for Gal-3 in liver pathology and fibrosis, here we show that Gal-3 is increased in the serum of patients with NASH.

CRP is a commonly used biomarker for systemic inflammation and has been found to be elevated in NASH [25]. There is evidence that serum CRP levels has a significant positive relationship with non-alcoholic fatty liver disease severity [26]. It has been suggested that CRP (and high sensitivity CRP) is an obesity independent marker of NASH [27,28,29]. However, in obese patients that were also diagnosed with NASH, CRP levels were increased in the liver and adipose tissue but not in serum [30]. A similar result was seen with high sensitivity CRP. Increased levels of high sensitivity CRP correlated with body mass index but did not predict the diagnosis of NASH [31,32]. There is also evidence that CRP is not indicative of NASH at all [33]. Similarly, we found no statistically significant difference in levels of serum CRP in NASH patients when compared to healthy controls.

The inflammasome has been found to play a role in NASH as well as other liver diseases. Inflammasome components have been proven to exist in several types of liver cells [10,11,12], strengthening the potential role of inflammasomes in liver inflammation. In hepatitis A, B, and C, there are elevated levels of IL-1β, thus indicating a potential role for the inflammasome [34]. In acetaminophen hepatotoxicity and ischemia/reperfusion, the NLRP3 inflammasome has been found to be involved in the inflammatory response [12,35]. The inflammasome has also been implicated in alcoholic liver disease (ALD), which encompasses a spectrum of liver diseases due to alcohol consumption. Accordingly, the effects of IL-1 on ALD have been associated to inflammasome signaling activation in bone marrow-derived Kupfer cells of mice [36].

One of the distinguishing features of NASH is inflammation. The inflammasome is thought to be responsible for this inflammation. For instance, ASC-deficient mice were protected from steatosis induced by a high fat diet [37]. Moreover, the NLRP3 inflammasome plays a major role in the inflammatory response in animal models of NASH. Accordingly, mRNA expression of NLRP3, ASC, caspase-1, pro-IL-1β, and pro-IL-18 is upregulated in the liver of NASH patients compared to healthy controls [10,13], and when NLRP3 was knocked out in a rodent model of NAFLD, the mice were protected from liver injury and fibrosis [13]. In the same study, knocking-in NLRP3 resulted in more severe inflammation and fibrosis. Evidence demonstrating the role of inflammasomes in NASH pathogenesis supports the idea that inflammasome components have potential as biomarkers of NASH. Here, we found that ASC and IL-18, in addition to Gal-3, can be used as reliable biomarkers of NASH based on AUC values of 0.73, 0.70, and 0.69, respectively.

Due to the beneficial role of a non-invasive diagnostic test for NASH, a few potential biomarkers have been studied. A serum biomarker test for cytokeratin-18 fragments (generated during cell death and apoptosis) has been tested but has a low sensitivity and has not been validated for NASH diagnosis [20]. High sensitivity CRP has also been tested as a serum biomarker with promising results [28,38], but as discussed above, CRP findings seem to vary across different cohorts of patients. Plasma Pentraxin-3 levels are increased in NASH patients, especially those with more severe disease (stages 3-4) compared to less severe disease (stages 0–2) and non-NASH (NAFLD patients) [39]. An in-depth analysis of plasma lipids proposed a signature of 20 candidates to distinguish NASH from steatosis [40]. Though most diagnostic tests use serum, one study evaluated urinary metabolomics to identify a panel of potential biomarkers to distinguish NASH from steatosis [41]. There are many potential biomarkers, for the diagnosis of NASH; however, none of them are ready for clinical use and all require further investigation [42].

Previous studies have shown AUC values of 0.63 for individual biomarkers such as fatty liver index [43] or 0.59 for tissue inhibitor of metalloproteinase 1 (TIMP 1), 0.73 for hyaluronic acid, 0.67 for cytokeratin-18, and 0.62 for human cartilage glycoprotein 39 (also known as YKL-40) [44] in individuals with NASH. In addition, combination of other diagnostic factors such body mass index, alanine aminotransferase, prolactin, high density lipoprotein cholesterol, and hemoglobin A1c provide AUC values of 0.86 [43]. Thus, our data showing single analytes with AUC values between 0.61 and 0.68 are consistent with other individual biomarkers of NASH, and together these findings highlight the difficulty in identifying individual biomarkers that are sensitive and specific enough for NASH. For instance, other studies have tested the effects of inflammation on liver elastography to obtain liver stiffness measurements and liver fibrosis in patients with hepatitis C virus (HCV) infections. Accordingly, in HCV, using models that included alanine aminotransferase (ALT), aspartate aminotransferase (AST), the AUC values were in the 0.9 range when measuring serum transaminase and liver fibrosis overestimation [45].

Increased inflammasome protein expression and Gal-3 have been associated with common comorbidities of NASH, such as hypertension, obesity, and type 2 diabetes, making it difficult to correlate these potential biomarkers with NASH exclusively [1,20,46]. Further studies analyzing serum samples from patients with varying NASH severities may help further confirm the role of ASC, IL-18, and Gal-3 as inflammatory biomarkers for this condition. Likewise, given that the presence of inflammation differentiates NASH from steatosis, further studies comparing serum levels of ASC, IL-18, and Gal-3 in patients with NASH to those with steatosis may help validate these potential biomarkers for NASH. Moreover, despite some of the comorbidities present in some of the patients used in this study, which are typical of patients with metabolic syndrome, the present study was powered to include patients with NASH and not the other comorbidities. Furthermore, logistic regression analyses were carried out to single out the diagnosis of NASH over other comorbidities; thus, providing evidence that the results obtained were more consistent with NASH than with the comorbidities that were presented by some of the patients in the study such as diabetes or hypertension. Accordingly, the *p*-values for the univariate logistic regression of NASH vs hypertension was *p* = 0.989, vs obesity *p* = 0.992, vs hypercholesterolemia *p* = 0.992, vs diabetes *p* = 0.988 and vs hyperlipidemia *p* = 0.988. Future studies are needed to validate ASC, IL-18, and Gal-3 as biomarkers of NASH in serum using a larger sample size for different disease severities. Most importantly, in this study, only some patients were diagnosed by biopsy procedures. Thus, additional studies are needed to validate these biomarkers in serum to correlate serum protein levels to protein levels obtained histologically from biopsy samples in all patients studied.

In conclusion, our results show that ASC, IL-18, and Gal-3 are elevated in the serum of patients with NASH. These three proteins present high AUC values, making them reliable biomarkers of NASH. Moreover, binomial logistic regression modeling using ASC, IL-18, and Gal-3 indicated that combined, these proteins are reliable to predict a diagnosis of NASH. These findings provide evidence for viable blood biomarker tests for NASH, which can comprise part of a biomarker panel to potentially eliminate the need for liver biopsy to diagnose this disease.

## 4. Materials and Methods

### 4.1. Participants

Serum samples were purchased from BioIVT (Hicksville, NY, USA). Donors were enrolled in the study Prospective Collection of Samples for Research sponsored by SeraTrials, LLC. with IRB number 20170439. Samples were obtained after informed consent. We analyzed serum samples from 14 male and 18 female patients diagnosed with NASH (Table 5) in the age range of 26 to 83 and 40 normal age-matched healthy controls. NASH diagnosis was made by a combination of methods, including magnetic resonance imaging, abdominal ultrasound, esophagogastroduodenoscopy, computerized tomography scans, fibroscan and biopsy as well as measurement of liver enzymes (ALT, AST), bilirubin, albumin and blood urea nitrogen.

### 4.2. Multi- and Single- Plex Assays

Protein concentrations for the inflammasome signaling proteins ASC and IL-18 as well as galectin-3 and C-reactive protein (CRP) in serum samples from NASH patients and age-matched controls was performed using the Ella System (Protein Simple, San Jose, CA, USA) as described in [47].

### 4.3. Biomarker Analyses

Data obtained by the Simple and Multi-Plex assays were analyzed with Prism 9 software (v. 9.0.0) (GraphPad). Outliers were removed and receiver operating characteristics (ROC) were calculated, obtaining a 95% confidence interval, a standard deviation and a *p*-value. A cut-off point was then obtained for a range of different specificities and sensitivities and their respective likelihood ratio and accuracy.

### 4.4. Statistical Analyses

After identifying and eliminating outliers using the robust regression and outlier removal (ROUT) method with a Q equal to 1%, normality was tested by the Shapiro-Wilk normality test, and statistical difference between groups was tested by the Mann-Whitney test for non-normally distributed data. A significant two-tailed p-value was considered at less than 0.05.

Binomial logistic regression models were compared for their ability to discriminate between patients diagnosed with NASH and those in the control group as well as for the calibration of the model for the goodness of fit through the AUC, the Akaike information criterion (AIC), the Bayesian information criterion (BIC), the McFadden-pseudoR^2^ and the Hosmer–Lemeshow goodness-of-fit test [48]. A multivariate binomial logistic model was chosen for variables with variables that univariately showed an AUC greater than 0.65 and a Hosmer–Lemeshow *p*-value greater than 0.05. The multivariate model was estimated through forward stepwise regression. The model chosen was the one that presented the lowest AIC.

## Figures and Tables

**Table 1:** ROC analysis results for signaling proteins in serum.

Biomarker	AUC	STD. Error	95% C.I.	*p*-Value
ASC	0.7317	0.05725	0.6195 to 0.8439	0.0004
IL-18	0.7036	0.05671	0.5924 to 0.8147	0.0016
Galectin-3	0.6891	0.06416	0.5633 to 08149	0.0064
CRP	0.5572	0.07247	0.4151 to 0.6992	0.4319

Control vs. NASH. AUC: Area under the curve; STD. Error: Standard Error; C.I.: Confidence Interval.

**Table 2:** Cut-off point analyses for signaling proteins in serum as markers of NASH.

Biomarker	Cut-Off Point (pg/mL)	Sensitivity (%)	Specificity (%)	PPV (%)	NPV (%)	Likelihood Ratio	Accuracy (%)
ASC	>394.9	81	60	55	84	2.051	68
IL-18	>269.2	77	60	51	83	1.952	66
Galectin-3	>7120	75	49	55	70	1.463	61
CRP	>2,895,004	68	42	52	58	1.177	55

Control vs NASH. PPV: Positive Predictive Value; NPV: Negative Predictive Value.

**Table 3:** Univariate analysis.

Analyte	ASC	IL-18	Galectin-3
Intercept (β_0_)	−2.764331	−2.620342	−2.661
Intercept (β_0_)—SE	0.683308	0.825223	8.415 × 10^−1^
Intercept (β_0_)—Significance	5.22 × 10^−5^	0.00150	0.00156
Coefficient (β_1_)	0.005215	0.008312	2.783 × 10^−4^
Coefficient (β_1_)—SE	0.001357	0.002724	9.267 × 10^−5^
Coefficient (β_1_)—Significance	0.000122	0.00228	0.00267
AUC	0.8106	0.7411	0.6882
McFadden Psuedo-R^2^	0.2783530	0.1264812	0.248
Hosmer–Lemeshow *p*-value	0.2872	0.3359	0.1248499
AIC	73.368	87.966	88.123
BIC	77.86482	92.46339	92.6202

SE: Standard Error; AUC: Aurea Under the Curve; AIC: Akaike information criterion; BIC: Bayesian information criterion.

**Table 4:** Multivariate analysis.

Analyte	ASC	IL-18	Galectin-3
VIF	1.091544	1.138538	1.060945
Coefficient	0.0036829	0.0055547	0.0002339
Coefficient—SE	0.0055547	0.0030760	0.0001259
Coefficient—Significance	0.012608	0.070949	0.063216
Intercept (β_0_)	−5.6164388		
Intercept(β_0_)—SE	1.5331389		
Intercept (β_0_)—Significance	0.000249		
AUC	0.71		
McFadden Psuedo-R^2^	0.3511984		
Hosmer–Lemeshow *p*-value	0.4867		
AIC	70.366		
BIC	79.35959		
Accuracy	0.7857143		

VIF: Variance Inflection Factor; SE: Standard Error; AUC: Aurea Under the Curve; AIC: Akaike information criterion; BIC: Bayesian information criterion.

**Table 5:** Patients with NASH.

NASH Patients	32
Sex	Males (44%)–Female (56%)
	14–18
Race	
Caucasian	22 (69%)
African	2 (6.25%)
Asian	3 (9.38%)
Mixed Race	3 (9.38%)
Other	2 (6.25%)
Age Rage	
Range	26–83
Median	61
Mean	57.13
Comorbidities	
HTN	20 (63%)
Obesity	12 (38%)
HCL	12 (38%)
Diabetes	11 (34%)
HLD	9 (28%)
CKD	4 (13%)
RA	2 (6.25%)

HTN: Hypertension; HCL: Hypercholesterolemia; HLD: Hyperlipidemia; CKD: Chronic Kidney Disease; RA: Rheumatoid Arthritis.

## Abbreviations

AIC	Akaike Information Criterion
ALD	Alcoholic Liver Disease
ASC	Apoptosis-associated Speck-like protein containing a Caspase recruiting domain
AUC	Area Under the Curve
BIC	Bayesian Information Criterion
CDAA	Choline Deficient Amino Acid-defined
C.I.	Confidence Interval
CKD	Chronic Kidney Disease
CRP	C-Reactive Protein
Gal-3	Galectin-3
HLD	Hyperlipidemia
HTN	Hypertension
IL	Interleukin
NAFLD	Non-Alcoholic Fatty Liver Disease
NASH	Non-Alcoholic Steatohepatitis
NPV	Negative Predictive Value
PPV	Positive Predictive Value
RA	Rheumatoid Arthritis
ROC	Receiver Operating Characteristic
VIF	Variance Inflection Factor
